# Row with the flow

**DOI:** 10.7554/eLife.03804

**Published:** 2014-07-29

**Authors:** Benjamin M Friedrich, Ingmar H Riedel-Kruse

**Affiliations:** 1 **Benjamin M Friedrich** is in the Max Planck Institute for the Physics of Complex Systems, Dresden, Germanyben@pks.mpg.de; 2 **Ingmar H Riedel-Kruse** is in the Department of Bioengineering, Stanford University, Stanford, United Statesingmar@stanford.edu

**Keywords:** flagella, synchronization, Volvox, flagellar beating, Volvox

## Abstract

Fluid forces are sufficient to keep flagella beating in synchrony.

**Related research article** Brumley DR, Wan KY, Polin M, Goldstein RE. 2014. Flagellar synchronization through direct hydrodynamic interactions. *eLife* **3**:e02750. doi: 10.7554/eLife.02750

**Image** The distance between two cells affects how well their flagella can synchronise their beating

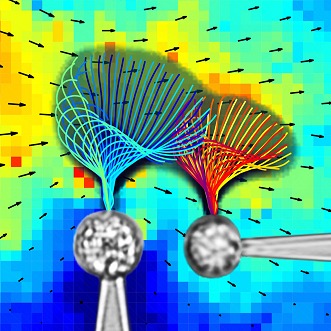

Cilia and flagella are found throughout nature. These slender cellular appendages—which range from a few micrometres to many millimetres in length—perform a wide range of roles in many different types of cells. They propel single-celled sperm and multi-celled algae through fluids, they direct chemicals called morphogens that are important for development in the growing embryo, and they pump mucus out of human lungs (Gray, 1928; Fliegauf et al., 2007). Many of these tasks involve a beating motion, which is driven by dynein motors inside the cilium or flagellum.

Cilia and flagella beat at a range of frequencies between once per second and 100 times per second. Two cilia never have exactly the same intrinsic beating frequency. Moreover, these frequencies randomly fluctuate during beating—this is known as noise. Nevertheless, thousands of cilia that are closely spaced on a surface can beat in synchrony to collectively perform a task, although this process is only partially understood.

Perhaps an obvious way cilia and flagella may be synchronised is through a central pacemaker inside the cells that controls the beating of several cilia on the same cell or tissue. However, this has been ruled out as even unconnected cells can synchronise their beating: for example, sperm cells can synchronise their flagella when swimming close together (Gray and Hancock, 1955; Riedel et al., 2005). Alternative sources of synchronisation control—including mechanical, chemical, or electrical signals—have been suggested, but the roles of each are still unknown. Now, in *eLife*, Raymond Goldstein and colleagues at the University of Cambridge—including Douglas Brumley as first author—report an elegant experiment that elucidates a physical mechanism that keeps cilia and flagella beating in time with each other (Brumley et al., 2014).

Previous experiments in which sperm were held in vibrating micro-needles showed that external physical forces can influence the intrinsic beating frequency of a single flagellum (Okuno and Hiramoto, 1976). The relationship between force and frequency has also been measured in green algal cells that were free to swim around normally (Geyer et al., 2013). Measuring how flagella respond to loads can provide insight on how the thousands of dynein motors inside the flagellum—each sensitive to external forces—work together to shape the beat.

Brumley et al. isolated two cells from the alga *Volvox*, with each cell containing (effectively) a single beating flagellum. Each cell was attached onto a separate micropipette. This prevented the cells from communicating with each other in any way other than through the fluid flows created by the beating flagella. It also allowed the researchers to experimentally vary the relative distance and orientation of both flagella. Provided this distance was small, the two beating flagella settled into a common rhythm. When the distance between the cells was increased, the synchronisation became imperfect—for example, one flagellum would sometimes add an extra beat—and eventually synchronisation broke down completely.

So how do multiple flagella synchronise? As each flagellum beats, it disturbs the liquid around it, generating a periodically varying fluid flow that exerts friction forces on other nearby flagella. This mechanically couples the flagella to each other. If this hydrodynamic coupling is strong enough to overcome both noise and the mismatch in the natural beating frequencies, then the flagella synchronise in a purely self-organised fashion.

Importantly, Brumley et al. determined exactly how the hydrodynamic coupling depends on the distance between flagella. The hydrodynamic phenomena experienced by beating flagella inside water are rather different to those experienced by a human swimming through water: indeed, they are akin to what a human would experience if they tried to swim through honey (Purcell, 1977). Theory predicts that the strength of the flow field around a beating flagellum is inversely proportional to the distance from the flagellum (Lauga and Powers, 2009). This is exactly what Brumley et al. measured, both when looking at an isolated flagellum, and also when examining how two flagella synchronise. Hence, the further apart two flagella are, the less force they apply on each other, eventually leading to a breakdown of synchrony. (Note, for freely moving cells, like swimming sperm cells, the flow field is instead inversely proportional to the square of the distance between the flagella, which can make quite a difference when trying to achieve synchronisation.)

Brumley et al. fitted their large experimental dataset to the corresponding mathematical model. This allowed them to determine quantitatively the coupling strength, frequency mismatch, and noise strength of two beating flagella. For example, the noise level is such that an isolated flagellum ‘forgets’ its initial phase after 30 beat cycles. This is consistent with recent direct measurements by ourselves and co-workers (Ma et al., 2014) and shows that fluctuations in the rate at which dynein motors work are perceivable at the scale of the flagellar beat.

Brumley et al. show that hydrodynamic forces alone are sufficient to cause cilia synchronisation, which could help to form a common quantitative understanding of how cilia and flagella interact across different scales (Figure 1). For example, how does the flagellum work internally? Brumley et al. show a force of 25 pN is produced in a stroke that is 10 µm long; therefore the hydrodynamic work done by a single flagellum can be estimated to be 25 pN*10 µm = 2.5*10^−16^ J per beat cycle. Interestingly, this value is of the same order of magnitude as the consumption of chemical energy by the 10,000 dynein motor heads inside the flagellum if each motor takes one step, burning one ATP molecule (each worth 10^−19^ J).

**Figure 1.**
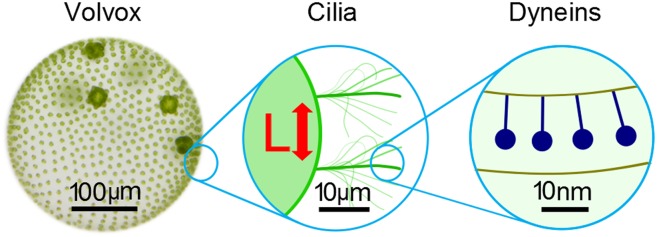
The beating of cilia and flagella can be considered at several different scales. Left: the green alga *Volvox* forms colonies that can contain thousands of cells. Each cell effectively has a single flagellum on its surface, and the flagella on different cells are able to beat in synchrony. Middle: pairs of cilia or flagella exert hydrodynamic forces on each other as they beat. Brumley et al. isolated two such flagella and varied the distance between them, L, to study the role of hydrodynamics in synchronisation. This revealed that the strength of the flow field, and hence the strength of synchronisation, is inversely proportional to L. Right: inside each flagellum, motor proteins called dyneins (dark blue) slide adjacent microtubules to drive the regular beat of the flagellum. The sensitivity of these motors to external forces may help cilia and flagella to synchronise.

FIGURE CREDIT: Frank Fox

Although these results show two cilia or flagella can be synchronised, how are thousands of them coordinated? Brumley et al. observe that synchrony breaks down at a distance of 10 µm, which is approximately the spacing between flagella on the surface of *Volvox*. Hence the coupling appears to be of the right strength to facilitate just nearest neighbour coupling. This is biologically advantageous because it is strong enough to enable synchrony, but not so strong as to generate large-scale phase locking. This, in turn, makes it possible for cilia and flagella to display more complex large-scale synchronisation phenomena, such as the metachronal waves that are essential for Volvox to propel itself along, or for ciliated airway epithelia to pump mucus away efficiently (Fliegauf et al., 2007).
